# Genetic Etiology of Renal Agenesis: Fine Mapping of *Renag1* and Identification of *Kit* as the Candidate Functional Gene

**DOI:** 10.1371/journal.pone.0118147

**Published:** 2015-02-18

**Authors:** Nyssa Becker Samanas, Tessa W. Commers, Kirsten L. Dennison, Quincy Eckert Harenda, Scott G. Kurz, Cynthia M. Lachel, Kristen Leland Wavrin, Michael Bowler, Isaac J. Nijman, Victor Guryev, Edwin Cuppen, Norbert Hubner, Ruth Sullivan, Chad M. Vezina, James D. Shull

**Affiliations:** 1 McArdle Laboratory for Cancer Research, Department of Oncology, School of Medicine and Public Health, University of Wisconsin, Madison, Wisconsin, United States of America; 2 Department of Genetics, Cell Biology and Anatomy, University of Nebraska Medical Center, Omaha, Nebraska, United States of America; 3 Hubrecht Institute, Utrecht, The Netherlands; 4 Max Delbruck Center for Molecular Medicine, Berlin, Germany; 5 Research Animal Resources Center, Graduate School, University of Wisconsin, Madison, Wisconsin, United States of America; 6 University of Wisconsin Carbone Cancer Center, School of Medicine and Public Health, University of Wisconsin, Madison, Wisconsin, United States of America; 7 Department of Comparative Biosciences, School of Veterinary Medicine, University of Wisconsin, Madison, Wisconsin, United States of America; Leibniz Institute for Age Research - Fritz Lipmann Institute (FLI), GERMANY

## Abstract

Congenital anomalies of the kidney and urogenital tract (CAKUT) occur in approximately 0.5% of live births and represent the most frequent cause of end-stage renal disease in neonates and children. The genetic basis of CAKUT is not well defined. To understand more fully the genetic basis of one type of CAKUT, unilateral renal agenesis (URA), we are studying inbred ACI rats, which spontaneously exhibit URA and associated urogenital anomalies at an incidence of approximately 10%. URA is inherited as an incompletely dominant trait with incomplete penetrance in crosses between ACI and Brown Norway (BN) rats and a single responsible genetic locus, designated *Renag1*, was previously mapped to rat chromosome 14 (RNO14). The goals of this study were to fine map *Renag1*, identify the causal genetic variant responsible for URA, confirm that the *Renag1* variant is the sole determinant of URA in the ACI rat, and define the embryologic basis of URA in this rat model. Data presented herein localize *Renag1* to a 379 kilobase (kb) interval that contains a single protein coding gene, *Kit* (v-kit Hardy-Zukerman 4 feline sarcoma viral oncogene homolog); identify an endogenous retrovirus-derived long terminal repeat located within *Kit* intron 1 as the probable causal variant; demonstrate aberrant development of the nephric duct in the anticipated number of ACI rat embryos; and demonstrate expression of *Kit* and Kit ligand (*Kitlg*) in the nephric duct. Congenic rats that harbor ACI alleles at *Renag1* on the BN genetic background exhibit the same spectrum of urogenital anomalies as ACI rats, indicating that *Renag1* is necessary and sufficient to elicit URA and associated urogenital anomalies. These data reveal the first genetic link between *Kit* and URA and illustrate the value of the ACI rat as a model for defining the mechanisms and cell types in which Kit functions during urogenital development.

## Introduction

Congenital anomalies of the kidney and urogenital tract (CAKUT) occur in approximately 0.5% of live births and together represent the most common class of developmental abnormalities in humans [1–4]. CAKUT is comprised of an assortment of interrelated phenotypes including bilateral renal agenesis (BRA), unilateral renal agenesis (URA), renal hypodysplasia, hydronephrosis, megaureter and pelviureteric junction obstructions. Together, these anomalies are the most frequent cause of end-stage renal disease in neonates and children [5,6]. The genetic bases of CAKUT are heterogeneous and only partially defined. Familial forms of CAKUT generally exhibit an autosomal dominant pattern of inheritance with incomplete penetrance [1,7]. Mutations in over 30 different genes have thus far been observed in association with CAKUT [1,4,8,9]. Roles for many of these CAKUT associated genes in urogenital development were first demonstrated in studies of genetically modified mouse models and the genes were subsequently implicated in the genesis of CAKUT by identification of mutations in families with multiple affected members. Other CAKUT associated genes were first identified in genetic studies of developmental syndromes that include anomalies in urogenital organs as associated phenotypes. Data from multiple genetic linkage and genome wide association studies, many of which were focused on vesicoureteral reflux as the phenotype of interest, further establish the heterogeneous genetic bases of CAKUT [6,10–16].

A solitary kidney resulting from URA or renal aplasia is a common CAKUT. A study in which 132,686 asymptomatic school children in China were evaluated by ultrasound and all suspected renal abnormalities were confirmed by radiography revealed a 0.08% incidence of solitary kidney and a 0.1% incidence of unilateral renal hypoplasia [17]. A similar study of 2920 asymptomatic 3 year olds in Japan revealed a 0.1% incidence of solitary kidney and a 0.07% incidence of unilateral renal hypoplasia [18]. A high incidence of solitary kidney has also been observed in adult populations examined postmortem. A solitary kidney was observed at an incidence of 0.09% in a large series of autopsies reported by the Armed Forces Institute of Pathology [19]. Similarly, a 0.18% incidence of solitary kidney was observed in a series of 13,775 consecutive autopsies performed at Vanderbilt University between 1928 and 1986 [20]. Multiple reports, only a few of which are cited here, indicate the occurrence of multiple cases of BRA and/or URA within families [21–24]. Moreover, the incidence of URA in first-degree relatives of individuals with BRA has been shown to significantly exceed the incidence of URA in the general population, strongly suggesting a genetic basis for familial renal agenesis. For example, Carter *et al*. observed BRA or URA in 7 of 199 (3.5%) siblings of individuals with BRA, whereas Roodhooft *et al*. observed asymptomatic URA in 3 of 71 (4.2%) parents and 2 of 40 (5.0%) siblings of cases of BRA [25,26]. It is becoming increasingly clear that the presence of a solitary kidney increases the risk of chronic kidney disease and hypertension and adversely impacts survival both in animal models and humans [5,27–30].

In order to understand more fully the genetic basis of renal agenesis, we are studying inbred ACI rats, which spontaneously exhibit URA and associated urogenital anomalies at an incidence of approximately 10%. The occurrence of URA in ACI rats was first reported in 1953 by Morgan and has been confirmed in multiple subsequent studies [31–38]. In addition to URA, female ACI rats generally exhibit an absent uterine horn ipsilateral to the missing kidney, whereas the ipsilateral vas deferens and epididymis of URA-affected males are frequently missing or incompletely developed [31–38]. Interestingly, the urogenital anomalies in ACI rats exhibit a pronounced right side bias. URA and associated urogenital anomalies are inherited as an incompletely dominant trait with incomplete penetrance in crosses between ACI and Brown Norway (BN) rats [39,40]. We mapped to a 14.4 megabase (Mb) interval on rat chromosome 14 (RNO14) a locus, designated *Renag1*, that appears to act as the sole genetic determinant of URA in reciprocal intercrosses between ACI and BN rats [39,40]. The goals of this study were to fine map *Renag1*, identify the causal genetic variant responsible for URA, confirm that the *Renag1* variant is the sole determinant of URA in the ACI rat, and define the embryologic basis of URA in this rat model. Data presented herein localize *Renag1* to a 379 kilobase (kb) interval that contains a single protein coding gene, *Kit* (v-kit Hardy-Zukerman 4 feline sarcoma viral oncogene homolog); identify the probable causal variant within *Kit* intron 1; demonstrate aberrant development of the nephric duct at day 11.5 of embryonic development; and demonstrate expression of *Kit* and Kit ligand (*Kitlg*) in the nephric duct.

## Results

### Fine mapping of *Renag1*

**Fig 1:**
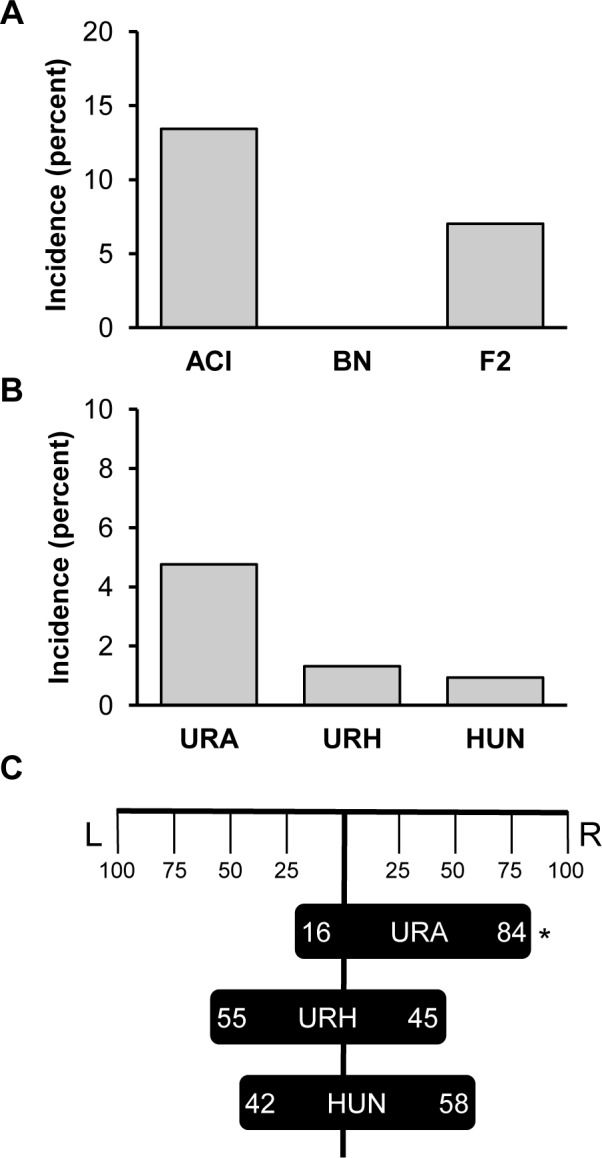
(BNxACI)F_2_ rats exhibit a diverse spectrum of urogenital anomalies. Urogenital anomalies were evaluated in a population of (BNxACI)F_2_ rats (n = 4994) that was generated by intercrossing BN females to ACI males. **A.** The incidence of all grossly discernable urogenital anomalies in (BNxACI)F_2_ rats is illustrated relative to that observed in ACI and BN rats. **B.** The incidence of unilateral renal agenesis (URA), unilateral renal hypoplasia (URH) and hydroureteronephrosis (HUN) in the (BNxACI)F_2_ population is illustrated. **C.** The frequencies of URA, URH and HUN observed on the left (L) versus right (R) sides are illustrated. An asterisk indicates statistically significant right side bias (*p* ≤ 0.05). Two (BNxACI)F_2_ rats exhibited bilateral HUN and were excluded from this analysis.

**Fig 2:**
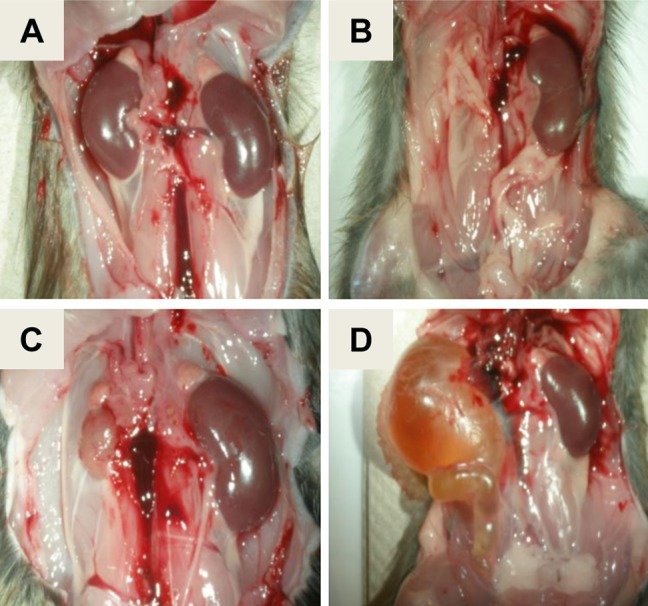
Representative renal anomalies observed in (BNxACI)F_2_ rats. **A.** Grossly normal renal development. **B.** Unilateral renal agenesis (URA). **C.** Unilateral renal hypoplasia (URH). **D.** Unilateral hydroureteronephrosis (HUN).

A population comprised of 4994 (BNxACI)F_2_ rats was generated and evaluated for renal abnormalities as a prerequisite for fine mapping *Renag1*. Of these F_2_ animals, 351 (7.0%) exhibited a renal abnormality, frequently in association with an abnormality in or absence of another urogenital organ(s) (Fig. 1A). The incidence of urogenital abnormalities observed in this F_2_ population was approximately half of that observed in ACI rats. These data are consistent with the published observation that URA and related urogenital abnormalities segregate as an incompletely dominant and incompletely penetrant trait in progeny generated by crossing ACI rats, which exhibit these developmental anomalies, and BN rats, which do not [40]. URA was observed in 238 of the 4994 F_2_ rats (4.8% incidence) and was the most frequently observed renal abnormality (67.8% of observed anomalies) in the F_2_ population (Fig. 1B). The other renal abnormalities observed in the F_2_ population were unilateral renal hypoplasia (URH), which was observed in 66 rats (1.3% incidence in F_2_ population, 18.8% of observed abnormalities), and an enlarged, fluid filled, vestigial kidney and ureter indicative of hydroureteronephrosis (HUN), which was observed in 47 rats (0.9% incidence in F_2_ population; 13.4% of renal abnormalities)(Figs. 1B and 2).

URA occurred on the right side in 200 of the 238 (84%) affected F_2_ rats, which constitutes a statistically significant right side bias (*p* = 2.2 x 10^−16^)(Fig. 1C). This observation is consistent with published reports on URA in ACI rats [35,38,40]. Interestingly, no statistically significant right or left side bias was evident in F_2_ animals that exhibited URH or HUN. A subset of the male F_2_ rats that exhibited URA also exhibited additional urogenital anomalies, including absent or incompletely developed ipsilateral adrenal gland, testis, vas deferens and/or epididymis. All of the females that exhibited URA lacked all or part of the ipsilateral uterine horn, 6 of the affected females lacked the ipsilateral ovary, and 5 lacked the ipsilateral ovary and adrenal gland.

**Fig 3:**
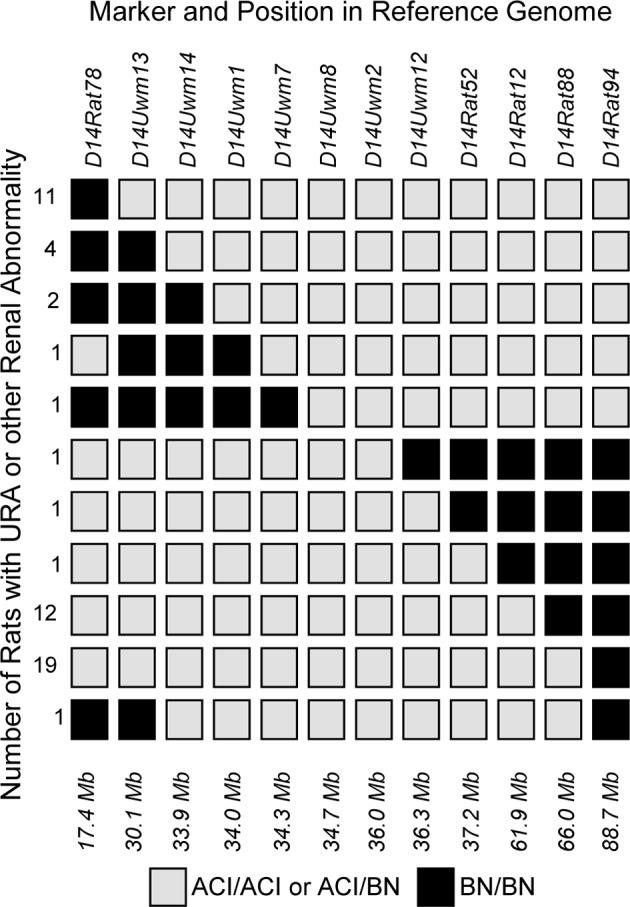
Fine mapping of *Renag1* through genotypic evaluation of (BNxACI)F_2_ progeny. Because a single ACI allele at *Renag1* is sufficient to elicit urogenital anomalies in progeny generated by crossing ACI and BN rats, homozygosity for BN alleles at an RNO14 marker in a (BNxACI)F_2_ rat affected by a urogenital anomaly excludes that marker from the *Renag1* locus. A total of 54 affected (BNxACI)F_2_ rats harbored recombinations within *Renag1* as defined previously and were thereby informative with respect to further refining the location of the *Renag1* causal variant. The markers genotyped and their locations along RNO14 are illustrated relative to genome assembly Rnor_5.0. Genotypes observed in the indicated numbers of affected (BNxACI)F_2_ rats are illustrated by the horizontal rows of boxes. Filled boxes represent those markers at which homozygosity for BN alleles was observed, thereby excluding that marker from *Renag1*. Open boxes represent markers at which homozygosity for ACI alleles or heterozygosity was observed. These data localize *Renag1* to the 2.0 Mb interval defined by *D14Uwm7* and *D14Uwm12*.

**Fig 4:**
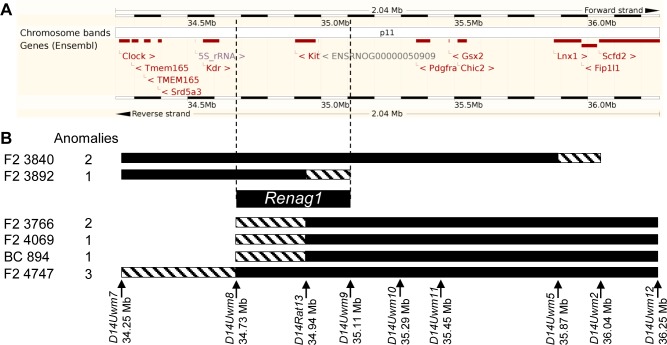
Fine mapping of *Renag1* through progeny testing. (BNxACI)F_2_ rats determined to harbor recombinations within the *Renag1* locus were mated to BN rats and the resulting progeny were evaluated for urogenital anomalies. Genotypes across RNO14 were determined for those progeny that exhibited a urogenital anomaly. Homozygosity for BN alleles at a marker excludes that marker from *Renag1*. **A.** The 2.0 Mb *Renag1* region (Ensembl genome browser, genome assembly Rnor_5.0) is illustrated. **B.** Six recombinant chromosomes were capable of eliciting urogenital anomalies in progeny generated by backcrossing the (BNxACI)F_2_ rats harboring those chromosomes to BN rats. The ACI segments of these chromosomes are illustrated as black bars, the segments of unknown genotype in which the recombinations occurred are illustrated by diagonal hatched bars, and the remaining segments of each chromosome are derived from BN rats. The identity of the rat of origin and the number of progeny exhibiting a urogenital anomaly are indicated to the left of each recombinant chromosome. The markers and genome coordinates at which genotypes were determined are indicated below the recombinant chromosomes. These data map *Renag1* to the 379 kb interval defined by markers *D14Uwm8* and *D14Uwm9*. This interval harbors a single protein coding gene, *Kit*.

Each of the 351 F_2_ rats that exhibited a renal abnormality was genotyped at polymorphic microsatellite markers distributed across the *Renag1* region on RNO14 to define more precisely the location of *Renag1*. The ACI allele of *Renag1* has been demonstrated to act in an incompletely dominant and incompletely penetrant manner to elicit URA; therefore, homozygosity for the BN allele at a marker excludes that marker from *Renag1* [40]. These analyses localized *Renag1* to the 2.0 Mb interval on rat chromosome 14p11 defined by markers *D14Uwm7* (34.25 Mb) and *D14Uwm12* (36.25 Mb) (Fig. 3). This region contains 12 annotated protein-coding genes and is orthologous to human chromosome 4q12 and mouse chromosome 5 (Fig. 4A).

Many of the male F_2_ rats that were generated in the experiment described above were genotyped at select markers on RNO14 prior to being euthanized. Those male F_2_ rats that harbored a recombination within the *Renag1* region were mated to BN females and the resulting progeny were evaluated for renal abnormalities. Five such recombinant chromosomes were capable of eliciting renal abnormalities in progeny that inherited the recombinant chromosome (Fig. 4B). In addition, one of the progeny generated by backcrossing male F_2_-4747 to BN rats exhibited URA and was determined to harbor a recombinant chromosome different than that harbored by F_2_-4747. Together, the genetic evaluation of progeny that inherited one of these six recombinant chromosomes and exhibited a renal abnormality further mapped the location of *Renag1* to the 379 kb interval on RNO14 defined by markers *D14Uwm8* (34.73 Mb) and *D14Uwm9* (35.11 Mb). This interval harbors a single annotated protein-coding gene, *Kit*.

### Confirmation of *Renag1* mapping to RNO14 through generation and evaluation of congenic rat strains

**Fig 5:**
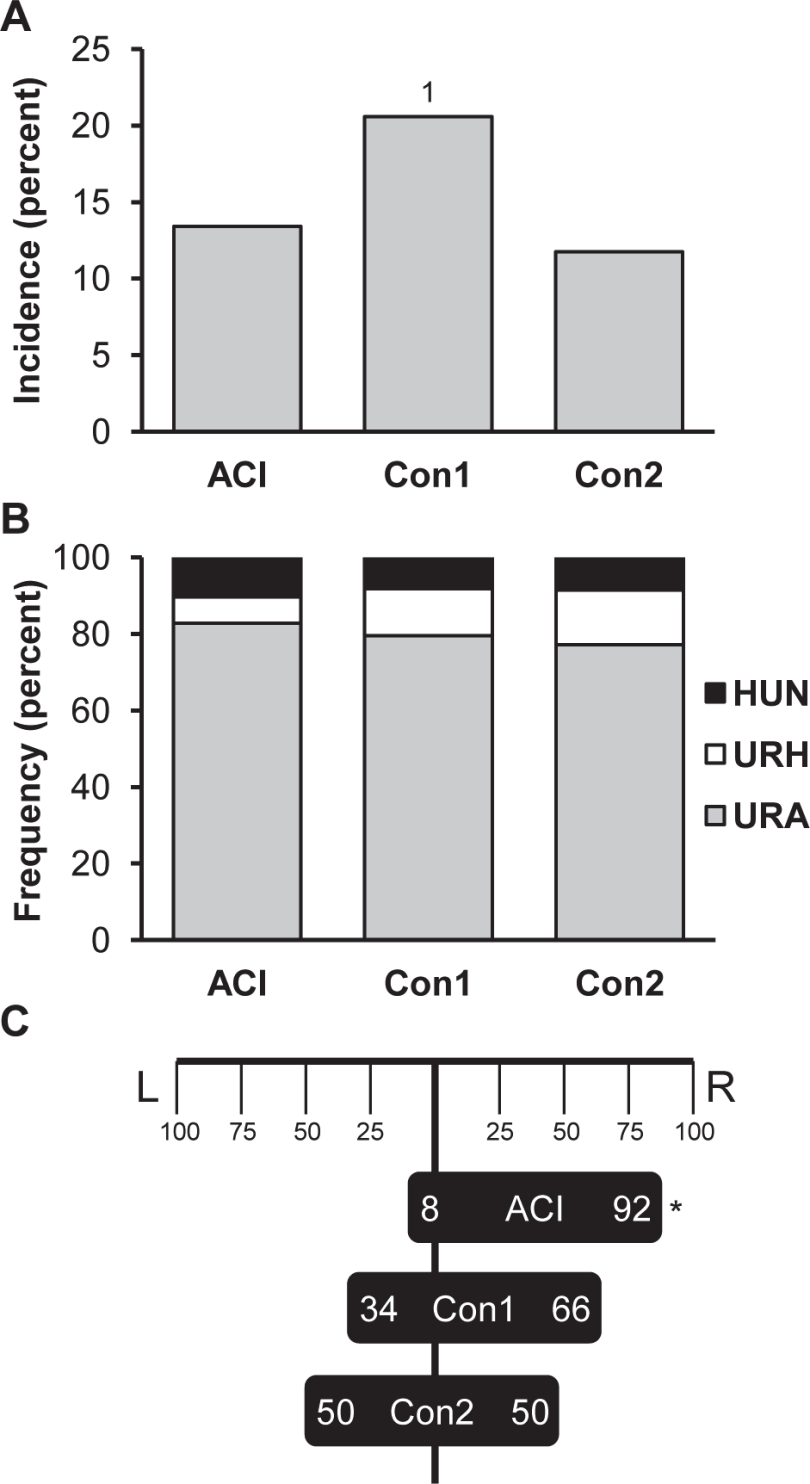
BN.ACI-Renag1 congenic rats exhibit the same spectrum and incidence of urogenital anomalies as ACI rats. The Con1 and Con2 congenic rat strains were generated as described in Materials and Methods. Each strain is homozygous for ACI alleles across the *Renag1* interval. **A.** The incidence of all grossly discernable urogenital anomalies in ACI (n = 216), Con1 (n = 238) and Con2 (n = 298) rats is illustrated. Numeral 1 indicates statistically significant difference relative to ACI. **B.** The frequency of unilateral renal agenesis (URA), unilateral renal hypoplasia (URH) and hydroureteronephrosis (HUN) in ACI, Con1 and Con2 rats is illustrated as a percent of total urogenital anomalies. **C.** The frequency of URA in ACI, Con1 and Con2 rats observed on the left (L) versus right (R) sides is illustrated. The asterisk indicates a statistically significant right side bias.

Two congenic rat strains, each of which harbors ACI alleles across *Renag1* on the BN genetic background, were generated as described below and evaluated with respect to urogenital anomalies. The BN.ACI-(*D14Uwm4*-*D14Rat39*) congenic strain (Con1) was generated from the recombinant chromosome that originated in rat F_2_-3766, whereas the BN.ACI-(*D14Uwm1*-*D14Uwm5*) congenic strain (Con2) was generated from the chromosome that originated in rat F_2_-3840 (Fig. 4B). Both Con1 and Con2 rats exhibited virtually the identical spectrum of urogenital abnormalities, including URA, URH and HUN, observed in ACI rats and (BNxACI)F_2_ rats. The overall incidence of urogenital abnormalities was 13.4, 20.6 and 11.7% in ACI, Con1 and Con2 rats, respectively (Fig. 5A). Interestingly, the incidence of urogenital anomalies in Con1 rats was significantly higher than in ACI (*p* = 0.0469) or Con2 (*p* = 0.0059) rats. URA was the most commonly observed urogenital anomaly in each rat strain, comprising 82.8, 79.6 and 77.1% of total urogenital abnormalities in ACI, Con1 and Con2 rats, respectively (Fig. 5B). URH comprised between 6.9 and 14.3% of total urogenital abnormalities and HUN comprised between 8.2 and 10.3% of total abnormalities in the three rat strains. These data indicate that homozygosity of ACI alleles at *Renag1*, when harbored on the genetic background of the BN strain, is sufficient to confer the full incidence and spectrum of urogenital abnormalities observed in ACI rats.

As expected from previous studies, URA in ACI rats was observed more frequently on the right side than the left; 91.7% right vs. 8.3% left (*p* < 0.0034, Fig. 5C). Interestingly, no right side bias was observed upon evaluation of URA in Con1 rats (*p* = 0.2452), Con2 rats (*p* = 1.000) or the combined congenic population (*p* = 0.3747). This observation indicates the right side bias observed in ACI and (BNxACI)F_2_ rats exhibiting URA is determined by a locus distinct from *Renag1*. The right-left distributions of URH and HUN were not analyzed statistically because of the low incidence of these anomalies in ACI, Con1 and Con2 rats. The incidence of renal abnormalities was similar in male and female ACI, Con1 and Con2 rats (data not shown).

### Cosegregation of urogenital anomalies and white spotting phenotype

**Fig 6:**
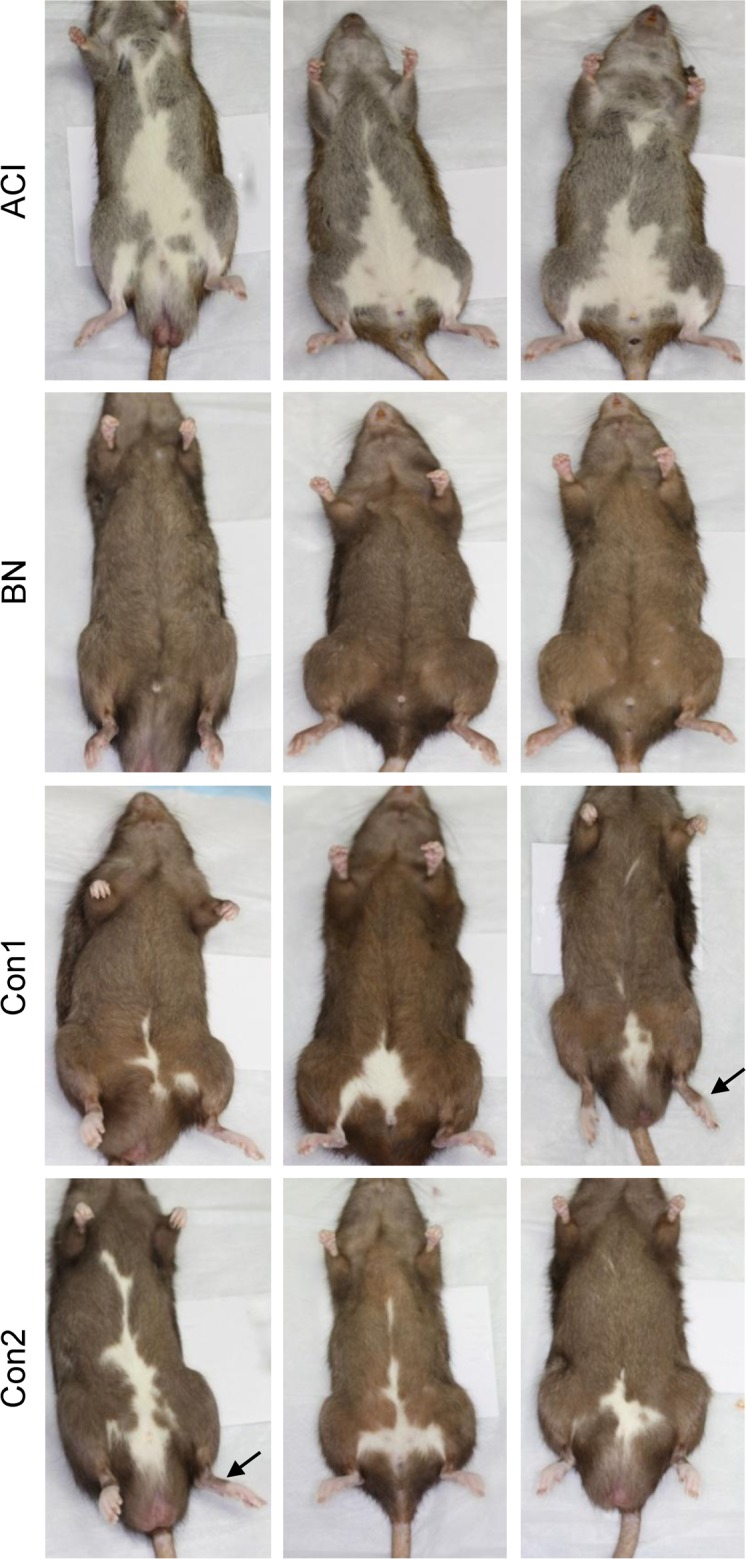
Abdominal white spotting is linked to the *Renag1* congenic interval. The ventral aspects of representative ACI, BN, Con1 and Con2 rats are illustrated. ACI rats harbor the *Irish* allele at the *Hooded* locus and exhibit abdominal white spotting. BN rats harbor the *Self* allele at *Hooded* and are fully pigmented on the abdomen. Con1 and Con2 rats harbor the ACI derived *Irish* allele at *Hooded* and exhibit varying amounts of abdominal white spotting and a clear demarcation of pigmentation on the rear feet (noted by arrows).

It became apparent during generation of the two congenic strains described above that the congenic intervals from the ACI strain that were being introgressed onto the BN genetic background harbored a genetic variant that confers a white spotting phenotype. Those progeny that inherited the recombinant chromosome at each generation of back crossing exhibited small patches of white on the abdomen. When heterozygous siblings were intercrossed, those progeny that were homozygous for the recombinant chromosomes (i.e., ACI alleles) exhibited a greater degree of abdominal white spotting. All individuals from each of the two congenic strains exhibited a varying degree of white spotting on the abdomen between the rear legs and extending cranially (Fig. 6). By contrast, BN rats only rarely exhibited any white spotting, and when present it was small and located on the chest, not the abdomen. Interestingly, the extent of the abdominal white spotting in the congenic animals was generally not as extensive as that exhibited by ACI rats. In addition, only the distal segment of each rear foot of the Con1 and Con2 rats was unpigmented, whereas the rear feet were entirely unpigmented in ACI rats. Together, these data indicate that a genetic variant inherited from the ACI donor strain and residing within the *Renag1* region on RNO14 acts in an incompletely dominant but completely penetrant manner to confer the unique pattern of white spotting observed in rats from the two congenic strains.

The *Hooded* locus, at which different alleles confer varying patterns of white spotting, has been mapped to intron 1 of *Kit* [41–43]. Moreover, associations between different *Kit* alleles and a wide variety of white spotting phenotypes have been noted in multiple species. ACI rats harbor the Irish allele, *h* ^*i*^, at *Hooded*, whereas BN rats harbor the Self allele, *H*, at *Hooded*, which explains the presence of abdominal white spotting in Con1 and Con2 rats. To further establish cosegregation of *Renag1* and *Hooded*, we analyzed unpublished data from previously described reciprocal intercrosses between ACI and Copenhagen (COP) rats that were performed to define the genetic bases of susceptibility to induction of mammary and pituitary tumors by administered estrogens [44–46]. Genome wide interval mapping analyses of the F_2_ progeny from these intercrosses linked URA to the *Renag1* region on RNO14 (data not shown). Forty F_2_ progeny from these intercrosses exhibited URA and each of these 40 affected F_2_ progeny harbored at least one ACI allele at *Renag1*, strongly supporting a genetic model in which an ACI allele at *Renag1* is both necessary and sufficient to confer URA in crosses between ACI and COP rats, similar to that observed in crosses between ACI and BN rats. Mapping by exclusion from *Renag1* of markers at which homozygosity for COP alleles were observed in URA affected F_2_ rats localized the causal genetic variant to the 48.55 Mb interval on RNO14 defined by markers *D14Rat78* (17.42 Mb) and *D14Rat88* (65.96 Mb) (data not shown). In addition, genome wide interval mapping analyses of the F_2_ progeny from these intercrosses between ACI (Irish allele, *h* ^*i*^, at *Hooded*) and COP rats, which harbor the Hooded allele, *h*, at *Hooded*, localized *Hooded* to RNO14. Fine mapping by inclusion within *Hooded* of markers at which homozygosity for COP alleles (*h* is recessive to *h* ^*i*^) was observed in those rats that exhibited the Hooded phenotype (white extending from abdomen upward on both flanks) further localized *Hooded* to the 20.72 Mb interval of RNO14 defined by markers *D14Arb6* (22.00 Mb) and *D14Rat15* (42.72 Mb)(data not shown). Together, these data indicate that *Renag1* cosegregates with allelic variants at *Hooded* that likely impact *Kit* expression in a cell type specific and/or temporal manner and thereby influence melanoblast migration during embryonic development resulting in variation in coat color.

### Sequence analyses of *Renag1*

In addition to our published and current data linking URA to the *Renag1* locus on RNO14 in multiple crosses between ACI rats and BN or COP rats, URA has been linked to a single marker on RNO14, *D14Rat65* (36.41 Mb), in a study of (F344 x ACI)F_2_ progeny evaluated in relation to prostate cancer susceptibility [47]. Based on the linkage and exclusion mapping data, we generated and/or evaluated available whole genome sequence data from the ACI, BN, COP and F344 rat strains to identify genetic variants within the 379 kb *Renag1* minimal interval that are unique to the ACI strain; i.e., variants where ACI rats differ from BN, COP and F344 rats. No such variants were identified when all available whole genome sequences for ACI (2 sequences), BN (2 sequences), COP and F344 rats were mapped onto Rat Genome Assembly 5.0 (Rnor_5.0) and compared (Table A in S1 File). The *Renag1* candidate gene, *Kit*, was also evaluated by standard PCR based sequencing methods (prior to the availability of whole genome sequences other than the BN reference sequence). The GenBank accession numbers for these *Kit* sequences are listed in Table B in S1 File. Five SNPs were identified upon comparison of *Kit* cDNA generated from mRNA prepared from ACI and BN rats. Four of these SNPs were synonymous variants within the *Kit* coding region: 1) nucleotide 34,947,632, exon 2, codon 51, glycine; 2) nucleotide 34,945,425, exon 3, codon 130, asparagine; 3) nucleotide 34,945,200, exon 3, codon 205, arginine; and 4) nucleotide 34,904,134, exon 21, codon 706, leucine. The exons harboring these SNPs were sequenced using DNA isolated from ACI, BN, COP and F344 rats, indicating that each of these four nucleotide variants present in ACI rats are also present in F344 and COP rats. These data are consistent with available whole genome sequence data for these four rat strains, with exception of the SNP at nucleotide 34,945,200, where one of two ACI sequences and the F344 sequence are instead identical to the reference BN sequence. The fifth SNP variant resides within the *Kit* 3’ untranslated region (UTR). Interestingly, the data generated by sequencing cDNA and exon 21 indicated that this variant was unique to the ACI rat strain. However, examination of available whole genome sequences at this position (nucleotide 34,903,999) indicated that one of the two available ACI sequences was consistent with data generated by sequencing cDNA and exon 21 whereas the other was identical to the reference BN sequence. In addition, the whole genome sequence for COP at this position differed from that observed upon sequencing COP cDNA and exon 21, and instead was identical to that observed upon sequencing cDNA and exon 21 from ACI rats. In spite of these inconsistencies between the whole genome and PCR based sequences, these data indicate that the five nucleotide variants residing within *Kit*, if they were to impact *Kit* mRNA stability or translation, are most probably not functionally associated with URA and associated urogenital anomalies.

**Fig 7:**
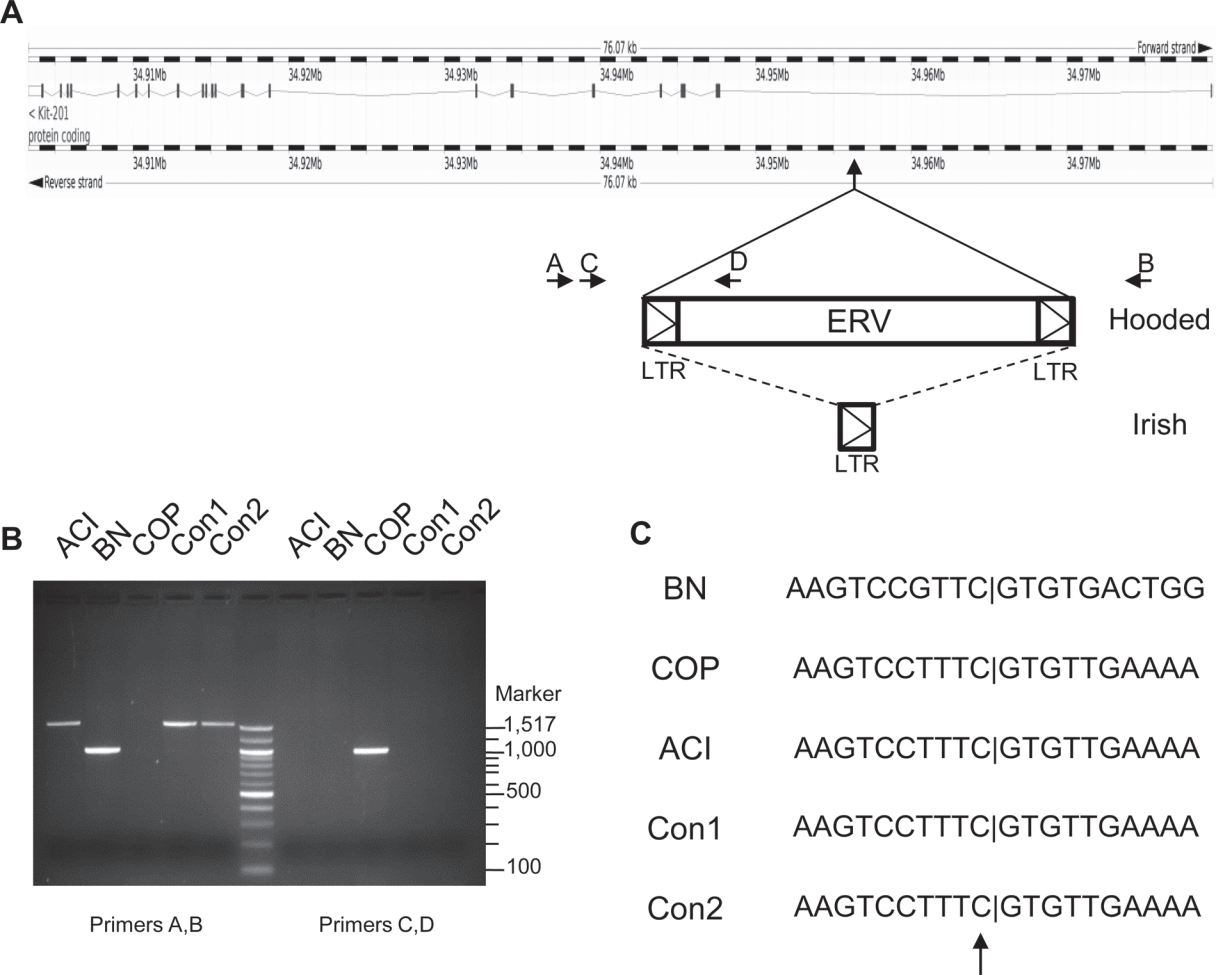
Genetic characterization of the *Hooded* locus in ACI, BN, COP, Con1 and Con2 rats. The Hooded coat color phenotype exhibited by COP rats results from the insertion of an endogenous retrovirus (ERV) into intron 1 of *Kit*. The Irish phenotype characteristic of ACI rats results from the presence of a solitary ERV long terminal repeat (LTR) at the same location within *Kit* intron 1. **A.** This panel illustrates the site and antisense orientation of the ERV insertion relative to *Kit*. Also illustrated are the relative positions of the sequences that correspond to the AB and CD primer pairs used to evaluate the site and type of ERV/LTR insertion. **B.** Left five lanes. The AB primer pair generated a 1006 bp PCR product from BN (*Self* allele at *Hooded*) genomic DNA template. The 1590 bp PCR product generated from ACI, Con1 and Con2 genome DNA is consistent with the presence of a solitary LTR within *Kit* intron 1 (*Irish* allele at *Hooded*). No product was generated when the AB primer pair was used to amplify genomic DNA from COP, due to the large size of the inserted ERV (*Hooded* allele). Right five lanes. The CD primer pair generated a 997 bp PCR product from COP (*Hooded* allele) genomic DNA. No product was generated from the CD primer pair upon amplification of genomic DNA from BN (*Self* allele), ACI, Con1 or Con2 (all *Irish* allele) rats, because primer D is within the inserted ERV element unique to the *Hooded* allele. Center lane. A 100 bp ladder (New England Biolabs) served as a marker for DNA fragment size. **C.** The illustrated nucleotide sequences flanking the ERV/LTR insertion site were confirmed by Sanger based capillary sequencing of PCR products generated using primer pair AB for BN, ACI, Con1 and Con2 rats, and primer pairs CD for COP rats. The upward arrow indicates the site of ERV insertion at nucleotide position 34,957,384 (rat genome assembly Rnor_5.0).

One genetic variant within the 379 kb *Renag1* interval that was not apparent upon evaluation of whole genome sequences mapped onto the Rnor_5.0 genome assembly is the rat strain specific presence of sequences related to a class I endogenous retrovirus (ERV) within intron 1 of *Kit* (at position 34,957,384 in Rnor_5.0). The ERV-related sequences were first identified by Kuramoto *et al*. as the causal variants residing within the *Hooded* locus, at which the Hooded (*h*) and Irish (*h* ^*i*^) alleles each confer distinct coat color phenotypes resulting from variation in the extent of melanoblast migration during embryogenesis [43]. These investigators demonstrated that BN, F344 and ACI rats each harbor a distinct allele at the *Hooded* locus: 1) BN rats harbor the *Self* allele (*H*) at *Hooded* and lack ERV-related sequences at the specific location in intron 1 of *Kit*; 2) F344 rats carry the *Hooded* allele (*h*) and harbor a 7098 bp ERV-related element, including both 5’ and 3’ long terminal repeats (LTR), within intron 1 of *Kit*; and 3) ACI rats carry the *Irish* allele (*h* ^*i*^) and harbor a 584 bp ERV-related element consisting of a single copy of the viral LTR. We have confirmed and extended the data of Kuramoto *et al*. by performing PCR based analyses across the site in *Kit* intron 1 into which the ERV-related elements would be inserted (Fig. 7). These data confirmed the lack of an integrated ERV in BN rats, illustrated the presence of the ERV in COP rats, which like F344 rats harbor the *h* (*Hooded*) allele at *Hooded*, and confirmed the presence of a single ERV LTR in ACI, Con1 and Con2 rats, which harbor the *h* ^*i*^ (*Irish*) allele at the *Hooded* locus (Fig. 7B). We further confirmed the insertion of the ERV related sequences by determining the nucleotide sequences across the insertion junction (Fig. 7C). Together, these analyses of sequence variants within the *Renag1* locus for BN, ACI, COP and F344 rats strongly suggest that the ERV-related LTR responsible for the Irish coat color phenotype in ACI rats is also the causal variant for URA and associated urogenital anomalies.

### Expression of *Pax2, Kit* and *Kitlg* in the nephric duct

**Fig 8:**
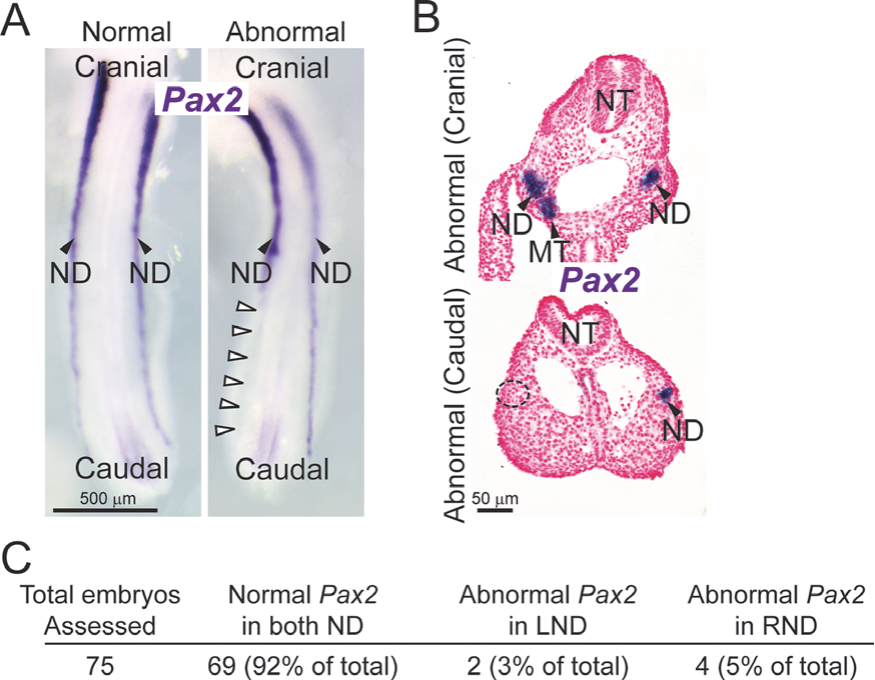
Embryologic basis of urogenital anomalies in ACI rats. e11.5 ACI rat embryos were stained by ISH to visualize mRNA expression (purple) of the nephric duct epithelium marker, paired box gene 2 (*Pax2*). The embryos were stained intact and the caudal portion was dissected after staining and positioned for optimal imaging. **A.** Representative images of each embryo’s caudal region revealed two different *Pax2* staining patterns: the normal pattern, where *Pax2* staining is present and extends to the cloaca in both nephric ducts, and the abnormal pattern, where *Pax2* staining in one nephric duct inappropriately terminates cranial to the hindlimb (indicated by white arrowheads). Note that in the same embryo, staining in the contralateral nephric duct extends past the caudal aspect of the hindlimb to the cloaca. **B.** Some whole-mount stained embryos were cut into transverse sections to reveal histological architecture. In most embryos, *Pax2* staining was identified in the bilaterally symmetrical nephric duct cell condensates. However, some embryos featured an atypical unilateral *Pax2* staining pattern. There was no histologically discernible cell condensate in the expected nephric duct region on the unstained side of the embryo (dashed circle). Abbreviations used are: ND, nephric duct; MT, mesonephric tubule; and NT, neural tube. Both images are the same magnification. **C.** Incidence of normal *Pax2* staining in both nephric ducts and abnormal (i.e., absent) staining in left and right nephric ducts of 75 rat embryos.

Pax2 is required for normal development of the nephric duct and is frequently used as a marker for identifying the nephric duct throughout the course of embryonic development. Therefore, expression of *Pax2* in the developing embryo was examined in order to understand better the developmental basis of urogenital anomalies in ACI rats. Evaluation of *Pax2* expression in e11.5 ACI embryos by *in situ* hybridization revealed strong labeling of the nephric duct and mesonephric tubules (Fig. 8). This pattern of *Pax2* expression was similar to that reported for e9.5 mouse embryos [48,49]. The majority of ACI embryos exhibited symmetrical *Pax2* expression in both the right and left nephric ducts (Fig. 8A, left panel). However, some embryos exhibited a clear unilateral absence of *Pax2* expression consistent with premature termination of the nephric duct (Fig. 8A, right panel). In a subset of these embryos, a pattern of intermittent *Pax2* expression was observed in the caudal segment of the nephric duct adjacent to the point of apparent truncation (data not shown). Select embryos were embedded in methacrylate polymer, sectioned and evaluated histologically. The sections selected for imaging were confirmed to be taken from a similar plane based on symmetry with respect to outline and position of nephric ducts, neural tube, caudal dorsal aortae and central gastrointestinal tract. These analyses confirmed the unilateral absence of *Pax2* expression in the expected anatomic location for the nephric duct (Fig. 8B). Additionally, no cell condensate in the expected location of the nephric duct was discernable upon histological examination, further associating lack of detectable *Pax2* expression with abnormal development of the nephric duct. Examination of 75 e11.5 ACI embryos revealed 6 embryos (8% incidence) with asymmetrical nephric duct *Pax2* expression (Fig. 8C). The absence of discernable *Pax2* staining was on the right side in 4 of these embryos and on the left side in two. Thus, the total incidence and right side bias of abnormal *Pax2* expression in the nephric duct closely resembled the incidence and asymmetry of urogenital anomalies exhibited by ACI rats.

**Fig 9:**
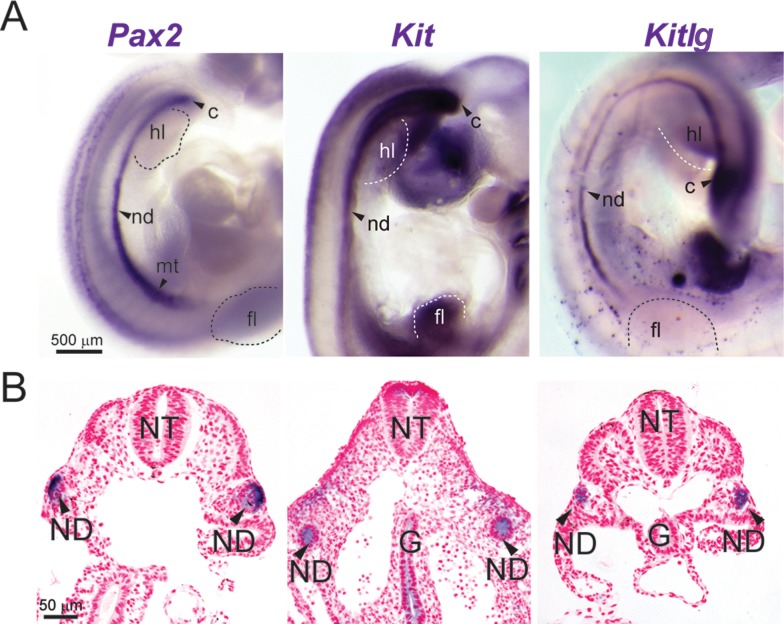
Kit and Kitlg mRNA are expressed in the nephric duct of ACI rat embryos. e11.5 ACI rat embryos were stained by ISH to visualize mRNA expression (purple) of the nephric duct epithelium marker *Pax2, Kit* (v-kit Hardy-Zuckerman 4 feline sarcoma viral oncogene homolog) or *Kitlg* (kit ligand). **A.** Lateral view of representative stained embryos. **B.** Some stained embryos were embedded in methacrylate and cut into transverse sections to reveal histological architecture. Abbreviations used are: C, cloaca; G, gut endoderm; FL, forelimb bud; HL, hindlimb bud; MT, mesonephric tubule; ND, nephric duct; and NT, neural tube. Images within each panel are of the same magnification and are representative of at least seven independent embryos.

Expression of *Kit* and *Kitlg* was similarly examined to begin to define the roles of these genes in urogenital development. *Kit* and *Kitlg* were observed to be expressed in the nephric duct of e11.5 ACI embryos evaluated by whole mount *in situ* hybridization (Fig. 9A). To confirm the localization of these mRNAs within and around the nephric duct, select embryos were embedded in methacrylate resin, sectioned, counter stained with nuclear fast red and evaluated. Histologic examination of embryo cross sections confirmed expression of both *Kit* and *Kitlg* mRNA in the nephric ducts (Fig. 9B). Together, these data indicate that URA and associated urogenital anomalies in the ACI rat are a manifestation of failed development of the nephric duct and strongly suggest a functional role of the Kit receptor and its cognate ligand in urogenital development in the rat.

## Discussion

The studies described herein localized the genetic variant responsible for URA and associated urogenital anomalies in the ACI rat to a 379 kb interval of RNO14 that harbors a single protein coding gene, *Kit*, as well as three predicted genes that may yield non-coding RNAs, *LOC102553597, LOC102553643* and *LOC102553856. Kit* encodes a transmembrane receptor tyrosine kinase that is activated by its cognate ligand Kitlg, also known as stem cell factor. Also presented herein are studies that indicated that *Kit* and *Kitlg* are expressed in the nephric duct of e11.5 ACI rat embryos. Published studies indicate that *Kit* is similarly expressed in the nephric duct of the developing mouse embryo [50,51]. These data suggest that perturbations in Kit signaling may contribute to urogenital abnormalities.

It has been suggested that failed development of the nephric duct gives rise to URA and associated urogenital anomalies in male and female ACI rats [35,36,40]. This study provides the first illustration of unilateral truncation of the nephric duct in a subset of ACI e11.5 embryos, as evidenced by *in situ* analyses of *Pax2* expression and histological evaluation. Roles for Kit signaling in migration, proliferation and/or survival of a variety of stem and progenitor cell types have been well documented [52]. Based on these data, we propose a model in which the causal *Renag1* genetic variant results in cell type specific and/or temporal alterations in *Kit* expression, and that this in turn impacts migration, proliferation and/or survival of a cell population(s) that is required for full and appropriately timed caudal extension of the nephric duct. Truncation of the nephric duct would account for the observed spectrum of aberrations in the ipsilateral urogenital tissues that are derived from the mesonephric duct, including vas deferens, seminal vesicle and epididymis, as well as the observed aberrations in the ipsilateral Mullerian duct derived tissues, such as the uterine horn. However, truncation of the nephric duct would not explain the absence of adrenal glands and gonads occasionally observed in ACI rats, suggesting that the *Renag1* causal variant may impact Kit signaling within multiple cell populations in the intermediate mesoderm. This model supports the hypothesis of Mackie and Stephens and is consistent with data from multiple studies that demonstrate that a specific gene mutation or environmental factor can exert pleiotropic actions on urogenital development resulting in a spectrum of distinct but related urogenital abnormalities [53–57].

To the best of our knowledge, this is the first study to suggest a causal role for a *Kit* allele in the etiology of URA and associated urogenital anomalies in any species. However, published reports provide circumstantial evidence linking *Kit* to aberrant urogenital development in humans. For example, the absence of the right kidney was noted in the child with piebaldism who was the subject of one of the first studies to associate that autosomal dominant trait to a loss of function mutation in *Kit* [58,59]. Second, agenesis/absence of the right kidney was noted in 2 of 12 cases of congenital abnormalities observed in offspring of women who were treated for chronic myelogenous leukemia during pregnancy with imatinib, a small molecule inhibitor of Kit and select other tyrosine kinases, including Pdgfra and Ret, which also play roles in renal development [60]. However, it is important to note that neither renal agenesis nor any of the other urogenital anomalies observed in ACI rats have been noted in studies of White Spotting (Ws) rats, which harbor a 12 nucleotide deletion in the *Kit* coding region that generates a dominant negative Kit protein, or in mice that harbor mutant *Kit* alleles [61–63].

The data from this study and our previous study indicate that the causal *Renag1* variant acts in an incompletely dominant and incompletely penetrant manner to confer development of urogenital anomalies. Interestingly, the same variant appears to act in an incompletely dominant but fully penetrant manner to confer the abdominal white spotting phenotype. We do not believe that these observations are contradictory. Instead, we interpret these data to suggest that the causal *Renag1* variant affects expression of *Kit*, which would then result in downstream actions on migration, proliferation and/or survival of specific populations of cells contributing to nephric duct development. If the number of cells required for the proper development of the nephric duct were not to achieve a critical threshold, duct elongation would fail at variable points along the cranial-caudal axis, resulting in the observed spectrum of anatomic abnormalities exhibited by rats harboring an ACI allele of *Renag1*. By contrast, variation in melanoblast migration resulting from actions of *Renag1* (i.e., *h* ^*i*^ allele at *Hooded*) on *Kit* expression would simply result in variation in the extent to which white spotting occurs.

A solitary ERV-derived LTR located within intron 1 of *Kit* is the sole variant within the *Renag1* locus that is unique to ACI rats. By contrast, BN rats lack ERV related sequences at the specified location in intron 1, whereas COP and F344 rats harbor the complete ERV at this location. This solitary LTR confers the Irish coat color trait upon ACI rats by means of its actions on *Kit* expression and melanoblast migration [43]. We hypothesize this LTR also confers the propensity for aberrant urogenital development upon ACI rats through its actions on *Kit* expression in an as yet unknown population(s) of cells. Additional studies are required to fully validate this hypothesis. The genomes of mammals harbor many thousands of full or partial copies of ERVs. It is well established that ERV LTRs can influence expression of nearby genes, and multiple examples are known in which LTRs have been exapted during evolution to function as cell type specific promoters or enhancers [64,65]. Also known are multiple examples in which ERVs and/or their LTRs are responsible for Mendelian or quantitative genetic traits [66–69].

The ACI rat is unique in that it is the only genetically defined rat model for studying the molecular and cellular bases of URA and associated urogenital anomalies. URA (i.e., solitary kidney) also segregates in heterogeneous stock (HS) rats, an outbred population derived from 8 inbred rat strains, including ACI [70,71]. Linkage of the solitary kidney phenotype to multiple loci was observed in a comprehensive QTL mapping study of HS rats, including the *Renag1* region on RNO14, suggesting the existence of modifiers of *Renag1* and/or other genes that independently contribute to urogenital anomalies in the HS rat population [71]. Moreover, a white spotting phenotype was mapped to the same region of RNO14 as the solitary kidney phenotype in the QTL mapping study of HS rats, suggesting that the Irish allele at the *Hooded* locus and *Renag1* cosegregate in the outbred HS rat population. The unilateral urogenital anomalies (UUA) rat represents another unique model for studying the etiology of urogenital abnormalities [72]. Although the spectrum of urogenital anomalies exhibited by UUA rats is similar to that of ACI rats, the urogenital anomalies occur exclusively on the left side in UUA rats, in contrast to the strong ride side bias observed for ACI rats.

This study demonstrated that a genetic variant in intron 1 of *Kit* is both necessary and sufficient to disrupt normal urogenital development in the ACI rat. The presented data strongly suggest that this variant ERV derived LTR exerts its actions through *Kit* expression and downstream signaling. If *Kit* is the gene through which the *Renag1* variant exerts its actions, then the incidence of URA and associated urogenital anomalies may be significantly higher in individuals with piebaldism than in the general population. Moreover, the spectrum of urogenital anomalies exhibited by ACI rats resembles the anomalies associated with specific genetic syndromes in humans. For example, the combination of right side preponderance of URA and an ipsilateral anomaly of the uterus exhibited by ACI rats resembles the phenotypic profile that is characteristic of Herlyn-Werner-Wunderlich (HWW) Syndrome [73–77]. Similarly, the anomalies exhibited by male ACI rats resemble those in humans with URA combined with congenital absence of the vas deferens [78–80]. An evaluation of the association of *Kit* and these genetic syndromes in humans appears warranted.

## Materials and Methods

### Housing and care of animals

The Institutional Animal Care and Use Committees of the University of Nebraska Medical Center (protocol 04-064-08) and the University of Wisconsin-Madison (protocol M02422) approved this entire study including all procedures involving live animals. ACI/SegHsd and BN/SsNHsd rats were obtained from Harlan Sprague-Dawley (Indianapolis, IN). Animals were housed under controlled temperature, humidity and 12h light/12h dark conditions in animal facilities that were accredited by the American Association for Accreditation of Laboratory Animal Care and operated in accordance with the standards outlined in *Guide for the Care and Use of Laboratory Animals*. Euthanasia prior to phenotypic evaluation was by asphyxiation with carbon dioxide.

### Fine mapping of *Renag1*

BN females were mated to ACI males to generate F_1_ progeny. F_1_ siblings were mated to generate F_2_ progeny. F_2_ progeny were euthanized at approximately 21 days of age and evaluated to ascertain the presence or absence of urogenital anomalies. Those animals that exhibited a urogenital anomaly were genotyped at a panel of microsatellite markers distributed across the *Renag1* locus on RNO14 as described previously [40,45,46,81]. Because an ACI allele at *Renag1* is required for URA and associated urogenital anomalies, homozygosity for the BN allele at a marker was interpreted to exclude that marker from the *Renag1* locus [40]. As fine mapping was in progress, a large number of male F_2_ rats were genotyped at RNO14 markers and those F_2_ rats that were determined to harbor recombinations within the *Renag1* locus were mated to BN females. The presence or absence of URA and associated anomalies was then evaluated in the resulting progeny in order to determine whether the specific recombinant ACI segment of RNO14 possessed the ability to elicit URA. Additional polymorphic microsatellite markers within the *Renag1* region were developed as needed. Pertinent information for these markers is included as Table C in S1 File.

### Generation and evaluation of *Renag1* congenic strains

The BN.ACI-(*D14Uwm4*-*D14Rat39*) congenic strain (Con1, Rat Genome Database id 8663453) was generated from the recombinant chromosome that originated in rat F_2_-3766, and the BN.ACI-(*D14Uwm1*-*D14Uwm5*) congenic strain (Con2, Rat Genome Database id 8663455) was generated from the chromosome that originated in rat F_2_-3840 using a selective breeding protocol adapted from that published previously [82–84]. The lineage carrying the recombinant chromosome from rat F_2_-3892 was lost during progeny testing, preventing development of a congenic strain that harbored that chromosome. Male [(BNxACI)F_1_ x BN] rats generated during progeny testing and known to harbor recombinant chromosomes capable of conferring URA were mated to female BN rats. The male N_3_ progeny from these matings were genotyped to identify those that were heterozygous across the *Renag1* region, the heterozygous males were backcrossed to BN females, and this process was repeated through multiple rounds of backcrossing. Rigorous negative selection to eliminate ACI alleles at markers on autosomes other than RNO14 was initiated at the N_5_ generation. The markers used for positive and negative selection during backcrossing are listed in Table D in S1 File. N_6_ progeny were evaluated in order to confirm inheritance of the propensity to exhibit urogenital anomalies together with ACI alleles at *Renag1*. After 7 generations of backcrossing for Con1 and 6 generations of backcrossing for Con2, a female rat from each strain harboring ACI alleles across *Renag1* was mated to a male BN rat to generate male progeny that were heterozygous at *Renag1* and carried the Y chromosome from the BN strain. Male rats from these matings were used in subsequent rounds of backcrossing to BN females. N_9_ siblings harboring the same recombinant chromosome were mated to generate founders for each congenic strain that were homozygous for ACI alleles at *Renag1* and homozygous for BN alleles at all background markers. Con1 and Con2 rats were generally evaluated for urogenital anomalies at approximately 21 days of age. Breeders from each congenic strain were evaluated upon retirement.

### Evaluation of linkage of *Renag1* and *Hooded* to RNO14 in reciprocal intercrosses between ACI and COP rats

Data on urogenital anomalies and coat color were collected as secondary phenotypes in previously described reciprocal intercrosses between ACI and COP rats that were performed to identify genetic determinants of susceptibility to estrogen-induced mammary and pituitary tumors [44–46]. These data were subjected to interval mapping and fine mapping analyses as described previously [40,44–46].

### Sequence evaluation of *Renag1* locus

Sequencing of the *Kit* candidate gene from ACI, BN, COP and F344 rats was performed using PCR amplified complementary DNA and/or genomic DNA as templates, standard Sanger sequencing methodologies, and an ABI 3730 capillary sequencing instrument. Sequence comparisons for the *Renag*1 interval from the same four rat strains were performed using whole genome sequence data from multiple sources available through the Rat Genome Database [85,86].

### Evaluation of site of ERV insertion in ACI, BN, COP, Con1 and Con2 rats

The sequences of the PCR primers used to amplify across the site of the ERV in *Kit* intron 1 were the same as those described by Kuramoto *et al*. and are presented in Table E in S1 File [43]. PCR products were analyzed on 1.2% agarose gels run in Tris acetate EDTA buffer. For sequencing, the PCR products were extracted from the gel using Qiagen QIAquick gel extraction reagents. Sequencing was performed in the University of Wisconsin Biotechnology Center on an ABI 3730XL capillary sequencer. The primers used for sequencing were the same as used for PCR amplification of genomic DNA template, plus additional nested primers designed to achieve full coverage of insertion junctions (Table E in S1 File).

### Evaluation of *Pax2, Kit* and *Kitlg* expression by *in situ* hybridization

Embryos were collected from ACI rats on day e11.5. Tissue collection, storage, and *in situ* hybridization (ISH) were performed as described previously [87,88]. The primers used to generate PCR-amplified probe templates from whole embryo rat cDNA were designed using Primer3 [89]. Primer sequences are presented in Table F in S1 File. A T7 RNA polymerase recognition sequence was incorporated onto the reverse primer for use in generating labeled RNA probes. The Primer Blast Program was used to ensure specificity of PCR primers for the target sequence [90]. Selected primer sequences uniquely match the target sequence and no other sequence in the rat reference genome. We used the MegaBLAST program to ensure specificity of the riboprobe sequence [91]. The riboprobe sequence was considered specific for its target when, using an EXPECT threshold of 0.01 and a word size of 128, it did not align with other members of the rat RefSeq RNA database. Embryos were processed together in a single tube for ISH and color development to allow for qualitative comparisons among them. BM Purple was used as alkaline phosphatase chromagen for digoxigenin- and fluorescein labeled riboprobe detection. The staining pattern for each riboprobe was assessed in at least seven litter-independent embryos. Embryos were stained intact. For some embryos, the caudal portion was dissected after staining and positioned for optimal imaging. Some whole-mount ISH stained samples were post-fixed in paraformaldehyde, embedded in JB-4 plus methacrylate polymer (Electron Microscopy Sciences, Hatfield, PA) and sectioned to a thickness of 5 μm with a rotary microtome. The sections were mounted on glass slides and counterstained with nuclear fast red.

### Statistical analyses of data

All categorical data were evaluated using Fisher’s exact test in R [92]. *p* values ≤ 0.05 were considered statistically significant.

## Supporting Information

S1 File Contains supporting information Tables A-F. Table A, Renag1 Variants. Genetic variants within the *Renag1* minimal interval. Table B, Kit GenBank. GenBank accession numbers for *Kit* sequences. Table C, Uwm Markers. Additional polymorphic microsatellite markers developed for genotyping within the *Renag1* region of RNO14. Table D, Congenic Markers. Markers used for positive and negative selection during generation of *Renag1* congenic strains. Table E, ERV Primers. Sequences of primers used to amplify and/or sequence across the site of the ERV insertion in *Kit* intron 1. Table F, ISH Oligos. Primers used to generate PCR-amplified probe templates for *in situ* hybridization.
(XLSX)
